# *CRKL*, *AIFM3*, *AIF*, *BCL2*, and *UBASH3A* during Human Kidney Development

**DOI:** 10.3390/ijms22179183

**Published:** 2021-08-25

**Authors:** Mirela Lozic, Luka Minarik, Anita Racetin, Natalija Filipovic, Mirna Saraga Babic, Katarina Vukojevic

**Affiliations:** 1 Department of Anatomy, Histology and Embryology, School of Medicine, University of Split, Šoltanska 2, 21 000 Split, Croatia; mirelalozic3@gmail.com (M.L.); luka.minarik@gmail.com (L.M.); anitamuic10@gmail.com (A.R.); filipovnatalija75@gmail.com (N.F.); msb@mefst.hr (M.S.B.); 2 Department of Medical Genetics, School of Medicine, University of Mostar, 88 000 Mostar, Bosnia and Herzegovina

**Keywords:** *CRKL*, *AIFM3*, *AIF*, *BCL2*, *UBASH3A*, kidney, development

## Abstract

We aimed to investigate the spatio-temporal expression of possible CAKUT candidate genes *CRKL*, *AIFM3*, and *UBASH3A*, as well as *AIF* and *BCL2* during human kidney development. Human fetal kidney tissue was stained with antibodies and analyzed by fluorescence microscopy and RT-PCR. Quantification of positive cells was assessed by calculation of area percentage and counting cells in nephron structures. Results showed statistically significant differences in the temporal expression patterns of the examined markers, depending on the investigated developmental stage. Limited but strong expression of *CRKL* was seen in developing kidneys, with increasing expression up to the period where the majority of nephrons are formed. Results also lead us to conclude that *AIFM3* and *AIF* are important for promoting cell survival, but only *AIFM3* is considered a CAKUT candidate gene due to the lack of AIF in nephron developmental structures. Our findings imply great importance of *AIFM3* in energy production in nephrogenesis and tubular maturation. *UBASH3A* raw scores showed greater immunoreactivity in developing structures than mature ones which would point to a meaningful role in nephrogenesis. The fact that mRNA and proteins of *CRKL*, *UBASH3A*, and *AIFM3* were detected in all phases of kidney development implies their role as renal development control genes.

## 1. Introduction

The permanent human kidney or metanephros appears around the 5th developmental week through the reciprocal interactions between the metanephric mesenchyme (MM) and the ureteric bud (UB). Under the inductive effect of the terminal branches of the UB (ampullae), the MM forms renal vesicles, which gradually differentiate into comma- and S-shaped bodies, and later on into more mature nephrons with Bowman’s capsules, glomeruli and nephron tubules that connect to the collecting system. Kidneys formed during the embryonic period grow and differentiate during the fetal period [1].

Kidney development can be divided according to the demeanor of the ampullae into four separate phases, in which they variably divide and induce nephrons. During the first phase (Ph1), which takes place from week 5 to around the 14th developmental week, each ampulla induces a single nephron. In phase two (Ph2) (15th to 20th–22nd developmental weeks), the ampullae divide sporadically but one ampulla is able to induce several nephrons which connect with collecting ducts into arcades. At the start of week 20–22, the kidney enters upon the third phase (Ph3) of development, when ampullae advance only by local cell proliferation. Finally, at around the 32nd–36th week, phase four (Ph4) is set in motion which is characterized by the disappearance of the ampulla, interstitial growth, and cell differentiation, which continues well into adult life [2,3]. During the first year of life, the glomerular filtration rate (GFR) greatly increases, due to changes in the glomeruli, which causes simultaneous changes in tubular transport for the preservation of glomerotubular balance necessary for the supply of nutrients to the growing infant [4].

The more complex a developmental process is, the greater the likelihood of abnormalities occurring in postnatal life. These abnormalities are described as congenital anomalies of the kidney and urinary tract (CAKUT). CAKUT are the most prevalent cause of end stage renal disease in children and they account for 20–30% of all prenatally diagnosed malformations. Today, over 80 genes are known to cause CAKUT when mutated, but the majority of genetic causes remain unknown [5,6]. Since there is a robust genetic influence on renal function and risk of disease, finding CAKUT candidate genes enables the development of new treatments and diagnostic procedures. In our study, we focus on *CRKL*, *AIFM3*, and *UBASH3A* as possible candidate genes in order to characterize their normal expression pattern during the four phases of kidney development.

The CRK like proto-oncogene, adaptor protein (*CRKL*) gene, is localized to chromosome 22q11.2 and has been previously linked to many signaling pathways in a wide variety of normal and diseased tissues [7,8,9,10]. *CRKL* regulates cell migration, morphology, differentiation, proliferation, apoptosis, and participates in intracellular signaling transduction from multiple growth factors, including the fibroblast growth factors (FGF), which are important for the normal development of the kidneys and urinary tract [11,12,13,14]. Most studies have analyzed the roles of the *CRKL* gene in cancer development, but its role in routine tissue development and non-cancerous states is equally important. Recent research has shown its implication in mediating tyrosine kinase signaling, and expression in the developing genitourinary (GU) tract in mice and humans [15]. Genetic disruption of *CRKL* contributes to the high incidence of GU defects associated with deletion at 22q11.2 [15]. In a previous study conducted by Lopez-Rivera et al., the role of the *CRKL* gene was explored on developing kidneys of humans, mice, and zebrafish. Molecular data obtained using whole exome-sequencing and targeted next-generation resequencing upholds the fact that haploinsufficiency of *CRKL* has a disruptive effect on renal development which supports the link between the *CRKL* gene and CAKUT. Furthermore, the patients with pathogenic mutations in the human homolog *CRKL* cause both syndromic and isolated congenital anomalies of the kidney and urinary tract [16]. Studies conducted on cultured podocytes with a specific deletion of *CRKL* found its association with altered podocyte process architecture and its primary involvement in regulating cell elongation [17,18].

Apoptosis inducing factor, mitochondria associated 3 (*AIFM3*) is an intracellular protein coding gene mapped to chromosome 22q11.21 flanking to the *CRKL* gene. Functions of AIFM3 include inducing apoptosis through a caspase dependent pathway, oxidoreductase activity, and reducing mitochondrial membrane potential [19,20]. RT-PCR and immunohistochemistry revealed its ubiquitous expression in human tissues [19]. A study by Murata et al. showed staining of AIFM3 in mitochondria and at a lower level in the endoplasmic reticulum (ER) membranes by confocal microscopy in HEK 293 cells, however, it is synthesized in the cytosol [21]. *AIFM3* overexpression has been shown to facilitate malignant transformation, development, and progression of breast cancer [22]. The expression of *AIFM3*, although present at very low levels in zebrafish pronephros, was seen in the urinary tract in human fetuses and children [16]. Genetic interaction studies using zebrafish suggested that haploinsufficiency of *CRKL* resulted in abnormal renal development, whereas deletion of its flanking genes, aifm3 and snap29, caused abnormalities only with co-suppression [16]. *AIFM3* together with *CRKL* is one of 10 candidate genes for classic bladder exstrophy (CBE), while Beaman et al. mapped their expression in developing kidneys and surrounding tissues [23]. RNA array data from embryonic mice detected Aifm3 in the embryonic bladder and CRKL in embryonic kidney mesenchyme and interstitium [24]. As of now, there are very few research articles on the topic of AIFM3, but its structural homolog AIF has been studied much more extensively.

The apoptosis inducing factor (*AIF*) has a locus on the X chromosome at position Xq26.1 and codes for the first identified pro-apoptotic protein that can trigger a unique caspase-independent cell death [25]. The AIF protein belongs to the same family as the aforementioned AIFM3 and they share mitochondrial localization as well as 35% homology, mainly in the a Pyr_redox_2 oxidoreductase domain (27%) that predicts a role in oxidative respiration [20]. AIF has been described as an essential redox-active enzyme under physiological conditions as a result of homozygous mouse mutants being embryonically lethal [26]. In humans, mutations in the *AIF* locus lead to symptoms such as axonal sensorimotor neuropathy, ataxia, deafness, mitochondrial encephalomyopathy, etc. [25]. Related diseases include Combined Oxidative Phosphorylation Deficiency [27], X-linked recessive Charcot-Marie-Tooth disease [28], and X-linked Deafness [29]. On the other hand, in pathological conditions it translocates from the mitochondria to the nuclei and mediates chromatin condensation and cell death [30]. Although its physiological role in metabolism is still poorly understood, it has been proposed that AIF serves as a redox switch which assesses metabolic conditions on the surface of the mitochondria and converts it into a life or death decision to purge metabolically compromised cells [31]. Despite the considerable research conducted on the gene in question, at present, this would be the first instance of its inquiry in renal development. Furthermore, our objectives included comparing the spatio-temporal patterning of the two family members to see if their physiological functions possibly aligned during kidney development.

B cell lymphoma 2, or *BCL2*, is a gene located on 18q21.33 and is best known for its regulation of the release of apoptosis-inducing factors [32]. BCL2 is essential for the normal development of the kidney although its role in renal disease is still under investigation. A BCL2 deficient phenotype generates such dramatic malformations due to augmented apoptosis leading to renal failure. Studies on the fetal kidney revealed that the expression of *BCL2*, as expected, does not follow the distribution of cells undergoing apoptosis [33,34].

Ubiquitin associated and SH3 domain containing A (*UBASH3A*) gene is mapped to chromosome 21q22.3 [35]. Its expression is thought to be restricted to lymphoid tissues and primarily T cells [36]. Genetic studies on mouse models revealed that simultaneous deletion of *Ubash3a* and *Ubash3b* causes hyperreactivity of T cells due to their involvement in the regulation of protein tyrosine kinase-mediated signaling [35]. UBASH3A participates in pro-apoptotic pathways by binding to AIF independently of caspase apoptotic events [37]. Jinwoo Ahn et al. found that *UBASH3A* was frequently mutated in patients with clear cell renal cell carcinoma and was deemed a metastasis-associated candidate gene [38]. Until now, *UBASH3A* has not been investigated on developing human kidneys.

The aim of this study was to investigate the expression and localization of CRKL, AIFM3, AIF, BCL2, and UBASH3A during normal human kidney development from the 13th week of development to the first year and a half postnatal, in order to determine which developmental period is most critical for the occurrence of CAKUT. Understanding the normal expression pattern of these genes can lead to better treatment within a precision medicine approach.

## 2. Materials and Methods

### 2.1. Tissue Procurement and Processing

Human specimens were collected after spontaneous abortions from the Department of Pathology at the University Hospital Center Split and were processed with the permission of the Ethical Committee of the University of Split, School of Medicine in accordance with the Helsinki Declaration (Williams 2008). External measurements (crown–rump length) and menstrual data were used to estimate the age of 14 fetuses (O’Rahilly 1972) between the 13th and 38th developmental weeks, first postnatal month, and 1.5 years of age. Two fetal kidneys belonged to Ph1 (13th week), four corresponded to Ph2 (16 th and 21 st week), two to Ph3 (27th and 29th week), and six to Ph4, which we divided into three sub phases: Ph4 prenatal (three fetuses, 37th and 38th week), three postnatal kidneys of which two were neonatal (Ph4 1 M), and one was one and a half years old (Ph4 1.5 Y). After the tissue samples were fixed using 4% paraformaldehyde in phosphate buffered saline (PBS) and dehydrated with graded ethanol dilutions, they were embedded in paraffin blocks and serially cut as 5 µm-thick sections, which were then mounted on glass slides. Prior to the application of immunofluorescence, proper tissue preservation was confirmed by hematoxylin and eosin staining of every tenth section.

### 2.2. Immunofluorescence

Following deparaffinization in xylol and rehydration in ethanol and distilled water, the mounted samples were steamed in a sodium citrate buffer for 30 min at 95 °C, as we described previously [39]. The following primary antibodies were used for immunofluorescence staining of individual samples: Rabbit anti-CRKL (1:50, HPA001100; Sigma-Aldrich, Darmstadt, Germany), rabbit anti-UBASH3A (1:800, sc-121; Santa Cruz, CA, USA), rabbit anti-AIFM3 (1:500, TA 306550; Origene, Rockville, MD, USA), sheep anti-AIF (1:500; AF5824, R&D Systems, Minneapolis, MN, USA). The following day sections were rinsed with PBS before incubation with the secondary antibodies Alexa Fluor 488 Donkey Anti-Rabbit, Rhodamine Red-X-conjugated AffiniPure Donkey Anti-Goat, (1:400, 711-545-152, 705-295-003; Jackson ImmunoResearch, Cambridge House, UK), for 1 h. Finally, sections were washed in PBS once more, nuclei were stained using 40,6-diamidino-2-phenylindole (DAPI) and then samples were cover-slipped (Immuno-Mount, Thermo Shandon, Pittsburgh, PA, USA). While being examined by a fluorescence microscope (Olympus BX51, Tokyo, Japan) equipped with a Nikon DS-Ri1 camera (Nikon Corporation, Tokyo, Japan), images were taken at ×40 magnification and processed using Adobe Photoshop. No staining was observed when sections were immersed in PBS rather than the primary antibodies.

### 2.3. Quantification and Statistical Analysis of Positive Cells

The applied protein markers presented with punctate and/or diffuse staining and any level of staining was regarded as positive, while lack of staining was regarded as negative. Structures analyzed were fully differentiated glomeruli (G), proximal convoluted tubules (PCTs), distal convoluted tubules (DCTs), immature glomeruli (IG), S-shaped bodies (SSBs), comma-shaped bodies (CSBs), and renal vesicles (RVs).

Quantitative analyses were performed as we described previously [40,41]. ImageJ software (National Institutes of Health, Bethesda, MD, USA) was utilized for cell counting (only positive structures were used for counting, if any were present) and from the data, percentages of positive cells per structure were obtained. The intensity of staining was classified into four grades: 0, implying the absence of any fluorescence: 1, weak; 2, moderate; and 3, strong. The raw scores were calculated by multiplying the percentage of positive cells within a certain kidney cortex structure (DCT, PCT, G, IG, SSB, CSB, RV) and its staining intensity. Results ranged from 0 to 300. At each time point, at least 20 structures were assessed. All data were expressed as mean ± SD and analyzed by the two-way ANOVA test with Tukey’s multiple comparisons test (GraphPad Software, La Jolla, CA, USA). The probability level of *p* < 0.05 was taken as statistically significant.

### 2.4. Data Acquisition and Statistical Analysis of Area Percentages

Images of the fetal and postnatal kidney cortex were taken at ×40 magnification, while being examined by a fluorescence microscope (Olympus BX51, Tokyo, Japan) equipped with a Nikon DS-Ri1 camera (Nikon Corporation, Tokyo, Japan), as we described previously [42]. To quantify CRKL, UBASH3A, AIFM3, and AIF immuno-expression, 15 non-overlapping representative visual fields of identical exposure time were captured for analysis. Green staining, be it punctate or diffuse, was interpreted as positive CRKL, UBASH3A, and AIFM3 immuno-expression, while red was considered positive for the expression of AIF. Analysis of CRKL did not include 1.5 year old specimens. ImageJ software (National Institutes of Health, Bethesda, MD, USA) was utilized for cell quantitative evaluation of immunoreactivity. Figures were prepared for analysis using subtraction of the median filter and color thresholding to measure the section percentage area covered by the positive signal. Co-localization of AIFM3 and AIF was calculated by dividing the area of overlap with the combined area of the two using Adobe Photoshop. GraphPad Software (GraphPad Software, La Jolla, CA, USA) was utilized for statistical analyses with the probability level of *p* < 0.05 being regarded as statistically significant. A one-way ANOVA test followed by Tukey’s post-hoc test was used to compare the immuno-expression in order to determine significant differences among phases of development.

### 2.5. RNA Isolation and qRT-PCR

DNA isolation was performed by affinity chromatography according to the Sigma-Aldrich protocol with the GenElute™ FFPE RNA Purification Kit. The RT-PCR analysis did not include 1.5-year-old specimens. The protocol starts by the process of deparaffinization of the formalin-fixed paraffin-embedded (FFPE) through a series of xylene and ethanol washes, followed by a digestion process with the provided Proteinase K and Digesstion Buffer A. Next, Buffer RL and ethanol are added to the lysate, and the solution is applied onto a spin-column. The bound RNA is washed off of the columns using Wash Solution A. After the RNA isolation, reverse transcription was performed using the High-Capacity cDNA Reverse Transcription Kit by Applied Biosystems. A master mix containing cDNA, selected forward and reverse primers (Table 1), SYBR green, and nuclease free water was mixed and put in a 96-well plate. All samples were done in duplicates, and RPS9 was used as a housekeeping gene. The negative control contained everything in the master mix, without the cDNA sample. The plate was then put into and analyzed by Applied Biosystems™ 7500 Real-Time PCR Systems.

## 3. Results

The expression of CRKL, AIFM3, AIF, and UBASH3A was analyzed on fully differentiated glomeruli (G), proximal convoluted tubules (PCTs), distal convoluted tubules (DCTs), immature glomeruli (IG), S-shaped bodies (SSBs), comma-shaped bodies (CSBs), and renal vesicles (RVs) of fetal and postnatal kidney samples by fluorescence microscopy. The tubules evaluated were localized to the superficial cortex and not from the juxtamedullary region. Results were expressed as area percentages of the positive signal as well as raw scores of all aforementioned structures and analyzed according to the four phases of kidney development as follows: Phase one (Ph1), phase two (Ph2), phase three (Ph3), phase four prenatal (Ph4 pre), phase four one month postnatal (Ph4 1 M), and phase four one and a half years postnatal (Ph4 1.5 Y). For statistical analyses, enough developmental structures (IG, SSB, CSB, and RV) were found only in the first three phases.

### 3.1. AIFM3

**Figure 1:**
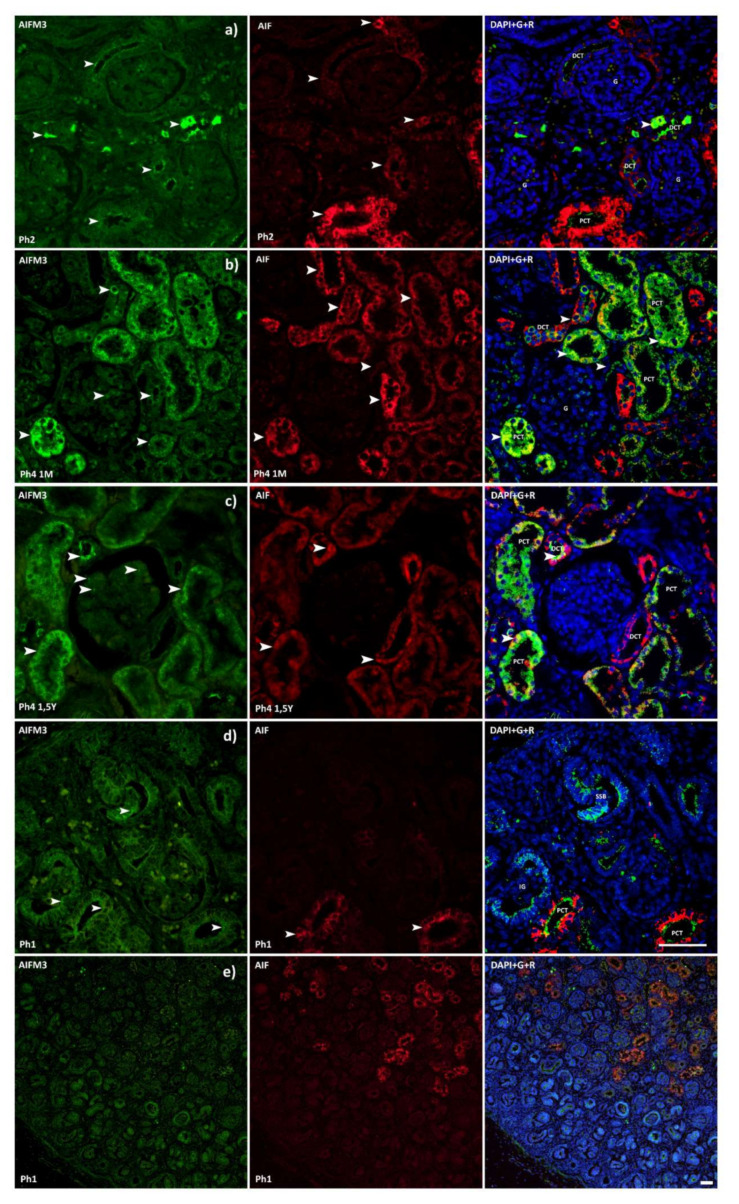
Immunofluorescence staining of human fetal (**a**,**c**,**d**) and postnatal (**b**) kidneys with the AIFM3 (green) and AIF (red) markers and their co-expression with DAPI nuclear staining. Expression of AIFM3 and AIF (arrows), glomeruli (G), proximal convoluted (PCT), distal convoluted tubules (DCT), immature glomeruli (IG), and S-shaped bodies (SSB). Co-expression of AIF and AIFM3 (arrows) can be seen on merged photographs as yellow. Images a, b, c and d were taken on magnification 40×, while e was taken on 10×. Scale bar is 40 μm. (**a**) The first three phases of kidney development mostly correspond regarding intensity and localization of AIFM3 thus a representative image was selected from Ph2. Individual cells of the DCTs exhibited a strong signal intensity. (**b**) Ph4 prenatal and Ph4 1M exhibited markedly more positive area percentage compared with the first three phase. Unlike the previous phases, a weak positive signal was detected in the glomeruli. Not much difference was observed regarding localization and signal intensity between the postnatal phases and the prenatal Ph4. (**c**) Ph4 1.5Y postnatal has significantly less positive area of tissue than both Ph4 pre and Ph4 1M, but the glomeruli displayed markedly more positive cells. (**d**,**e**) The developing structures of the nephron were devoid of AIF signal in all phases where they were present, unlike AIFM3 where they were all markedly more positive than the mature structures.

**Figure 2:**
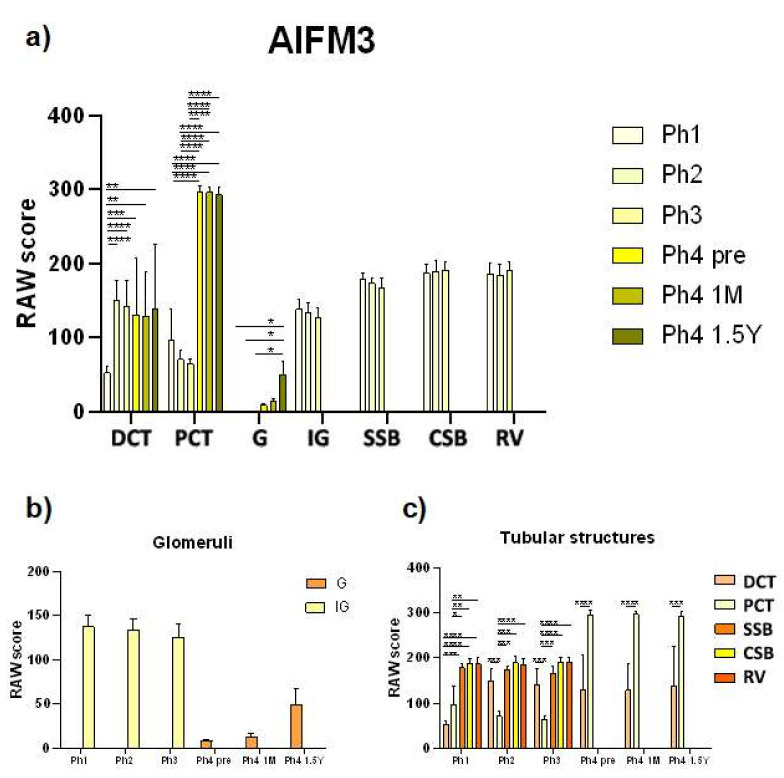
RAW scores of AIFM3 in the cortical nephron structures of human fetal and postnatal kidneys. Fully differentiated glomeruli (G), proximal convoluted tubules (PCT), distal convoluted tubules (DCT), immature glomeruli (IG), S-shaped bodies (SSB), comma-shaped bodies (CSB) and renal vesicles (RV), phase one (Ph1), phase two (Ph2), phase three (Ph3), phase four prenatal (Ph4 pre), phase four one month postnatal (Ph4 1 M), and phase four one and a half years postnatal (Ph4 1.5 Y). For statistical analyses, enough developmental structures (IG, SSB, CSB, and RV) were found only in the first three phases. Data are presented as the mean ± SD (vertical line) and analyzed by the two-way ANOVA test with Tukey’s multiple comparisons test. Significant differences were indicated by * *p* < 0.05, ** *p* < 0.01, *** *p* < 0.001, **** *p* < 0.0001. (**a**) Comparison of structures from different periods. (**b**) Comparison of mature and immature glomeruli from the same period. (**c**) Comparison of DCT, PCT, SSB, CSB and RV from the same period.

**Figure 3:**
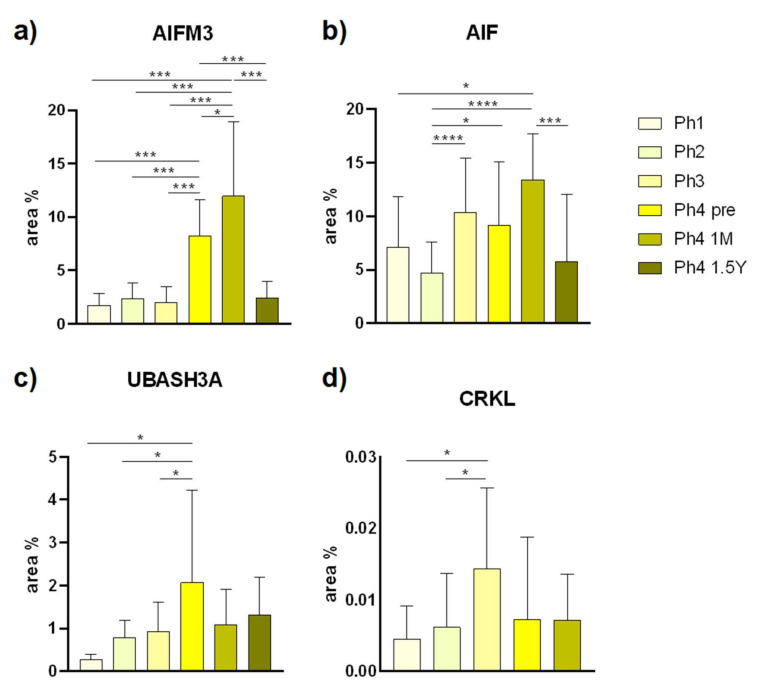
The area percentages of AIFM3 (**a**), AIF (**b**), UBASH3A (**c**), and CRKL (**d**) in human fetal and postnatal kidney cortex. Phase one (Ph1), phase two (Ph2), phase three (Ph3), phase four prenatal (Ph4 pre), phase four one month postnatal (Ph4 1 M), and phase four one and a half years postnatal (Ph4 1.5 Y). Data are presented as the mean ± SD (vertical line) and analyzed by the one-way ANOVA test with Tukey’s multiple comparisons test. Significant differences were indicated by * *p* < 0.05, *** *p* < 0.001, **** *p* < 0.0001.

**Figure 4:**
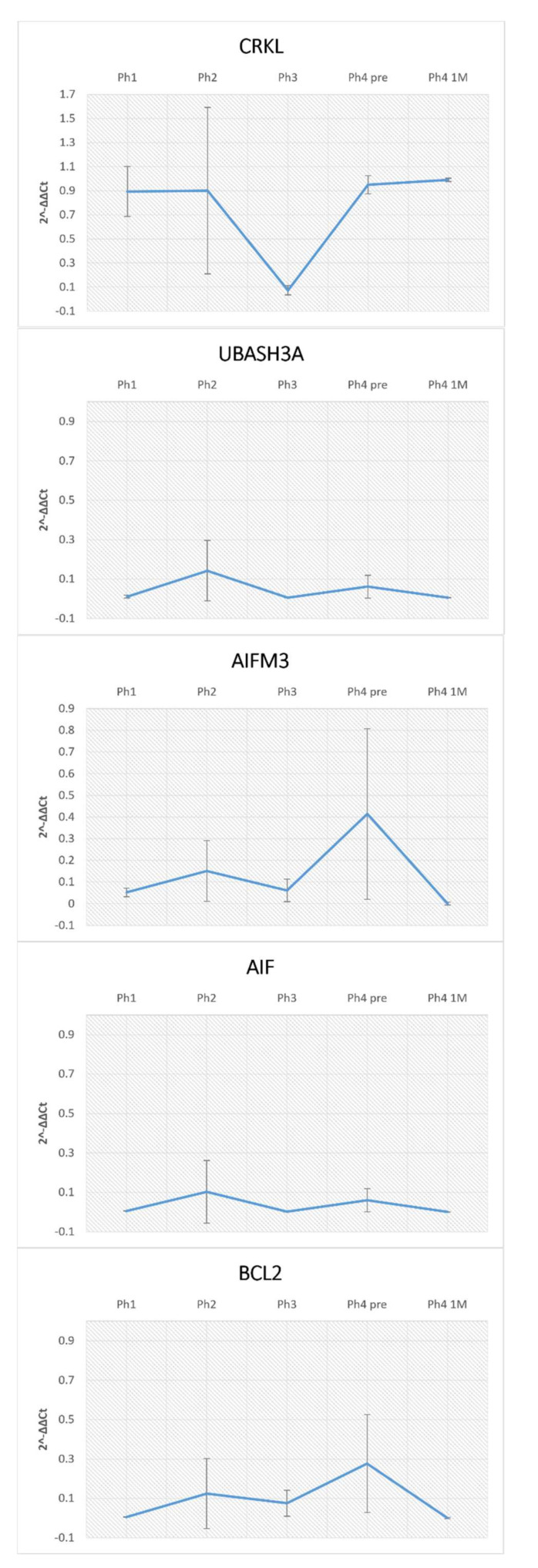
The qRT−PCR analysis of human fetal and postnatal kidneys using primers for CRKL, UBASH3A, AIFM3, AIF, and BCL2 and their calculated 2^-ΔΔCt^.

Analysis of AIFM3 revealed diffuse cytoplasmatic as well as luminal staining (Figure 1). Samples belonging to Ph1 displayed that the staining intensity was classified as moderate. Localization was luminal in developing structures, DCTs and PCTs. The raw score of the Ph1 DCTs was significantly lesser compared to all the following phases (Figure 2). The second and third phase of kidney development mostly corresponds to Ph1 regarding intensity and localization. Individual cells of the DCTs exhibited a strong signal intensity. The first three phases, compared with Ph4 prenatal and Ph4 1 M, exhibited markedly less positive area percentage (*p* < 0.001) (Figure 3). Corresponding to that, the PCTs of those three phases were significantly less positive (*p* < 0.0001) than those of Ph4, be it pre or postnatal (Figure 2). In Ph4 prenatal, unlike the previous phases, a weak positive signal was detected in the glomeruli. Moreover, the PCTs showed strong diffuse cytoplasmatic staining. Not much difference was observed in regards to localization and signal intensity between the postnatal phases and the prenatal Ph4. However, a significant difference (*p* < 0.05) was found in the evaluation of area percentage, where Ph4 1 M had a considerably larger percentage mean (Figure 3). Although the distribution and intensity of immunofluorescence mostly matches the other phase four groups, the Ph4 1.5 Y postnatal has significantly less positive area of tissue (*p* < 0.001) than both Ph4 pre and Ph4 1 M (Figure 3). Be that as it may, the glomeruli displayed markedly more positive cells (*p* < 0.05) in this, the oldest analyzed kidneys (Figure 2). Apart from Ph1, we found significant differences between DCTs and PCTs in all phases. In Ph2 and Ph3, PCTs had notably less immuno-expression (*p* < 0.001), while contrarily all Ph4 PCT raw score means far surpassed DCTs (*p* < 0.0001 in Ph4 pre and Ph4 1 M; *p* < 0.001 in Ph4 1.5 Y) with them being as large as 296.7 (Figure 2). Concerning the structures of the developing nephron, they were all markedly more positive than PCTs in the first three phases where they are most present and more positive than DCTs only in Ph1 (Figure 2). The qRT-PCR analysis revealed the highest expression of mRNA in Ph4 1 M, corresponding to the immunofluorescence results (Figure 4).

### 3.2. AIF

**Figure 5:**
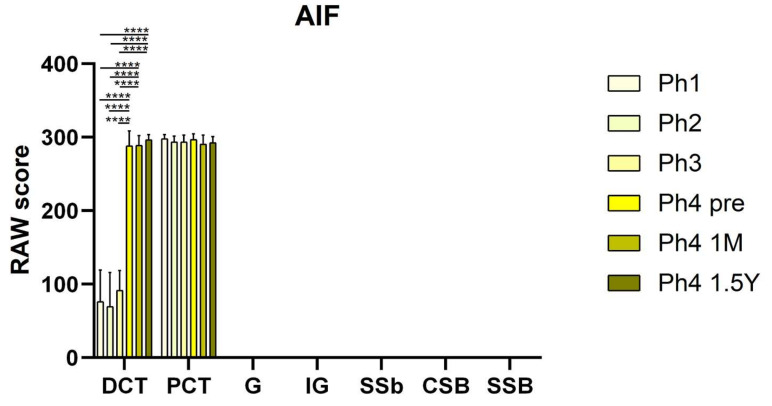
RAW scores of AIF in the cortical nephron structures of human fetal and postnatal kidneys. Fully differentiated glomeruli (G), proximal convoluted tubules (PCT), distal convoluted tubules (DCT), immature glomeruli (IG), S-shaped bodies (SSB), comma-shaped bodies (CSB) and renal vesicles (RV), phase one (Ph1), phase two (Ph2), phase three (Ph3), phase four prenatal (Ph4 pre), phase four one month postnatal (Ph4 1 M), and phase four one and a half years postnatal (Ph4 1.5 Y). For statistical analyses, enough developmental structures (IG, SSB, CSB, and RV) were found only in the first three phases. Data are presented as the mean ± SD (vertical line) and analyzed by the two-way ANOVA test with Tukey’s multiple comparisons test. Significant differences were indicated by ** *p* < 0.01, *** *p* < 0.001, **** *p* < 0.0001.

AIF positive cells were recognized as red staining in the cytoplasm (Figure 1). In Ph1 and Ph2, the PCTs had strong immunoreactivity in the cytoplasm, while DCTs had moderate staining in the apical parts of the cell membranes. However, in Ph3 and Ph4 prenatal, the DCTs had a similar distribution of immuno-reactivity as the PCTs in this and the previous phase. Results revealed a significantly lesser area percentage of Ph2 than Ph3 (*p* < 0.001), Ph4 prenatal (*p* < 0.05), and Ph4 1 M (*p* < 0.001) (Figure 3). Additionally, Ph4 1 M had a markedly greater area of positivity than Ph1 (*p* < 0.05) and the oldest analyzed samples of Ph4 1.5 Y (*p* < 0.001) (Figure 3). Cell counting and signal intensity evaluation revealed significantly greater raw scores (*p* < 0.0001) of DCTs belonging to prenatal and postnatal Ph4 than all the earlier phases (Figure 5). Unlike AIFM3, the developing structures of the nephron were devoid of signal in all the phases where they were present (Figure 1). The qPCR analysis confirmed the above-mentioned area percentage results pattern of expression (Figure 4).

**Figure 6:**
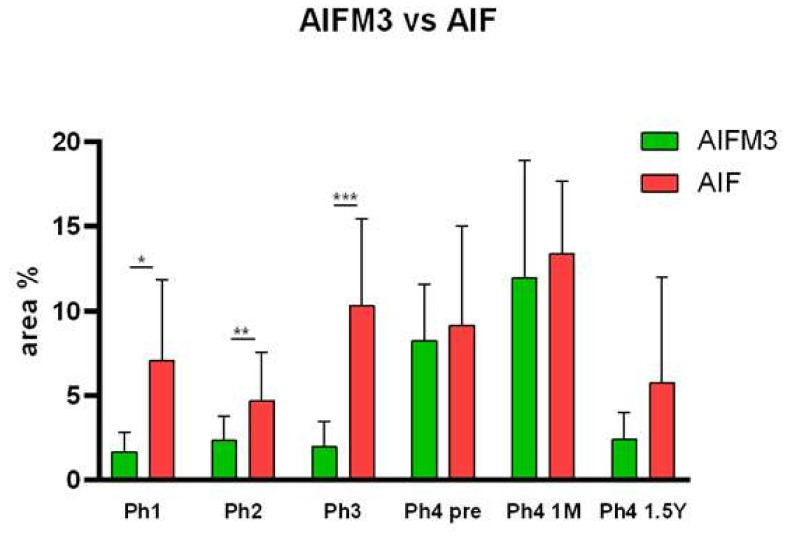
The area percentages of AIFM3 and AIF in human fetal and postnatal kidney cortex side by side comparison. Phase one (Ph1), phase two (Ph2), phase three (Ph3), phase four prenatal (Ph4 pre), phase four one month postnatal (Ph4 1 M), and phase four one and a half years postnatal (Ph4 1.5 Y). Data are presented as the mean ± SD (vertical line) and analyzed by the two-way ANOVA test with Tukey’s multiple comparisons test. Significant differences were indicated by * *p* < 0.05, ** *p* < 0.01, *** *p* < 0.001.

**Figure 7:**
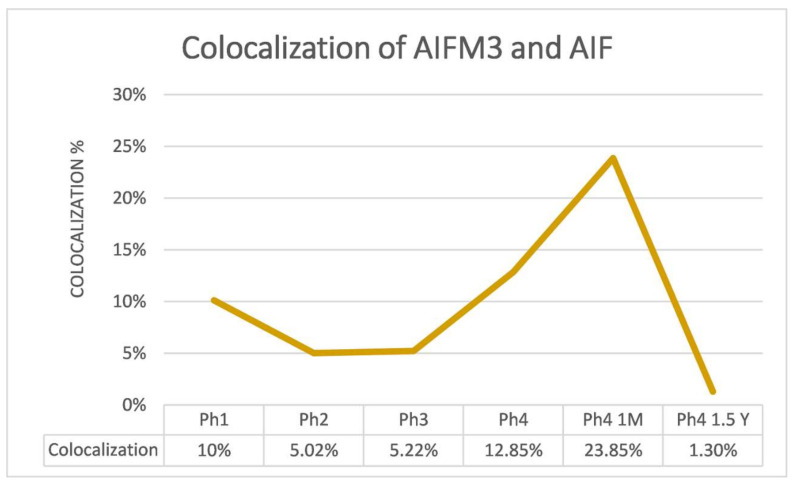
Colocalization values presented as percentages in graph and table form. Phase one (Ph1), phase two (Ph2), phase three (Ph3), phase four prenatal (Ph4 pre), phase four one month postnatal (Ph4 1 M), and phase four one and a half years postnatal (Ph4 1.5 Y).

Since we were interested in the discrepancies between AIFM3 and AIF, a two-way ANOVA analysis was conducted with their area percentage data. Results show considerably more of the AIF protein in Ph1 (*p* < 0.05), Ph2 (*p* < 0.01), and Ph3 (*p* < 0.001) (Figure 6). By implementing double immunofluorescence, we were able to calculate the colocalization of AIFM3 and AIF in different phases, which ranged from 23.85% in Ph4 1 M to a mere 1.3% in the following Ph4 1.5 Y (Figure 7). The qRT-PCT data were used to perform a two-way ANOVA test with Tukey’s multiple comparisons test, which revealed no significant differences between AIFM3 and AIF mRNA (Figure 4).

### 3.3. UBASH3A

**Figure 8:**
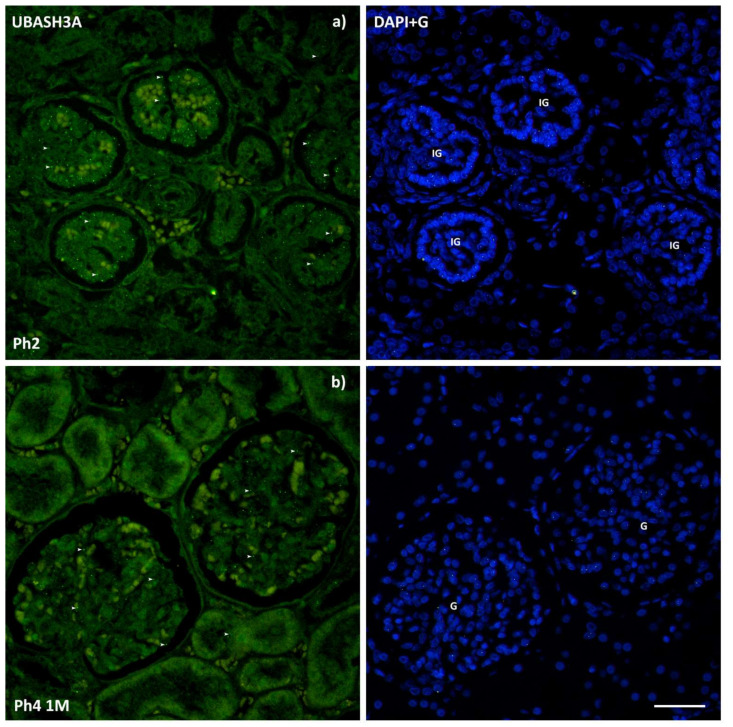
Immunofluorescence staining of human fetal (**a**) and postnatal (**b**) kidneys with the UBASH3A (green) marker and the isolated signal with DAPI nuclear staining. Expression of UBASH3A (arrows), glomeruli (G), and immature glomeruli (IG). Significant differences in immunoreactivity were found between fully differentiated (**b**) and immature (**a**) glomeruli. Images (**a**,**b**) were taken on magnification 40×. Scale bar is 40 μm.

**Figure 9:**
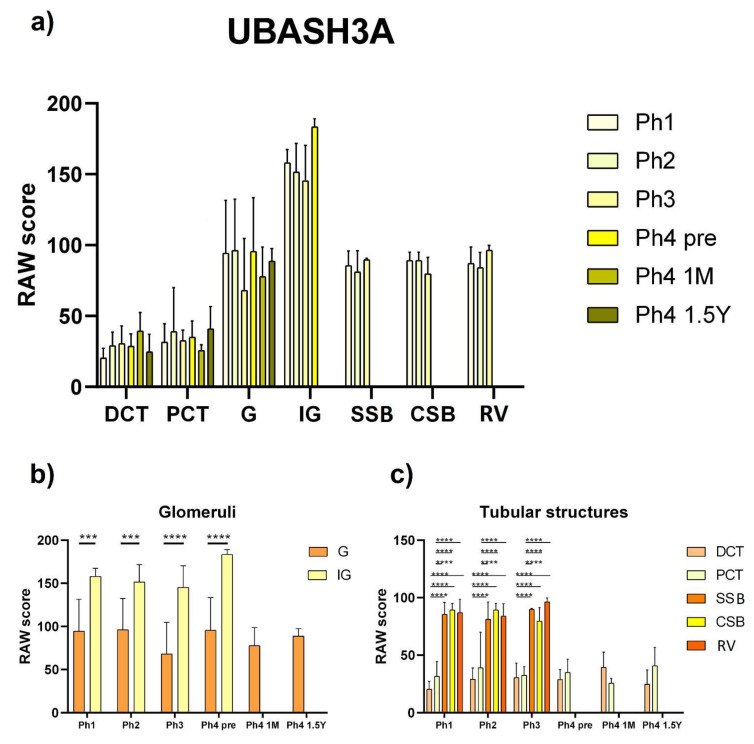
RAW scores of UBASH3A in the cortical nephron structures of human fetal and postnatal kidneys. Fully differentiated glomeruli (G), proximal convoluted tubules (PCT), distal convoluted tubules (DCT), immature glomeruli (IG), S-shaped bodies (SSB), comma-shaped bodies (CSB) and renal vesicles (RV), phase one (Ph1), phase two (Ph2), phase three (Ph3), phase four prenatal (Ph4 pre), phase four one month postnatal (Ph4 1 M), and phase four one and a half years postnatal (Ph4 1.5 Y). For statistical analyses, enough developmental structures (IG, SSB, CSB, and RV) were found only in the first three phases. Data are presented as the mean ± SD (vertical line) and analyzed by the two-way ANOVA test with Tukey’s multiple comparisons test. Significant differences were indicated by ** *p* < 0.01, *** *p* < 0.001, **** *p* < 0.0001. (**a**) Comparison of structures from different periods. (**b**) Comparison of mature and immature glomeruli from the same period. (**c**) Comparison of DCT, PCT, SSB, CSB and RV from the same period.

The UBASH3A protein was observed as punctate, green, mostly nuclear staining. The intensity of tubular staining was weak, while the glomeruli, regardless of maturity, demonstrated a moderate fluorescence signal (Figure 8). Concerning the area percentage, the prenatal Ph4 was markedly increased (*p* < 0.05) compared to the first three phases. Aside from that, there were minor shifts in expression ranging from a mean of 0.28% in the first phase to 2.07% in the aforementioned prenatal Ph4 (Figure 3). No significant differences were detected in the analysis of raw scores of structures in different phases. However, Sidak’s multiple comparisons test uncovered variable immunoreactivity between fully differentiated and immature glomeruli (*p* < 0.001 in Ph1 and Ph2, *p* < 0.0001 in Ph3 and Ph4 pre) in phases where both were sufficiently present. Correspondingly, the MM derived developmental structures (SSB, CSB, and RV) were markedly more positive (*p* < 0.0001) that the mature tubules (DCT and PCT) (Figure 9). The qPCR analysis did not show any significant differences between phases, but the fetal tissue used included the medulla of the kidneys, not just the cortex (Figure 4).

### 3.4. CRKL

**Figure 10:**
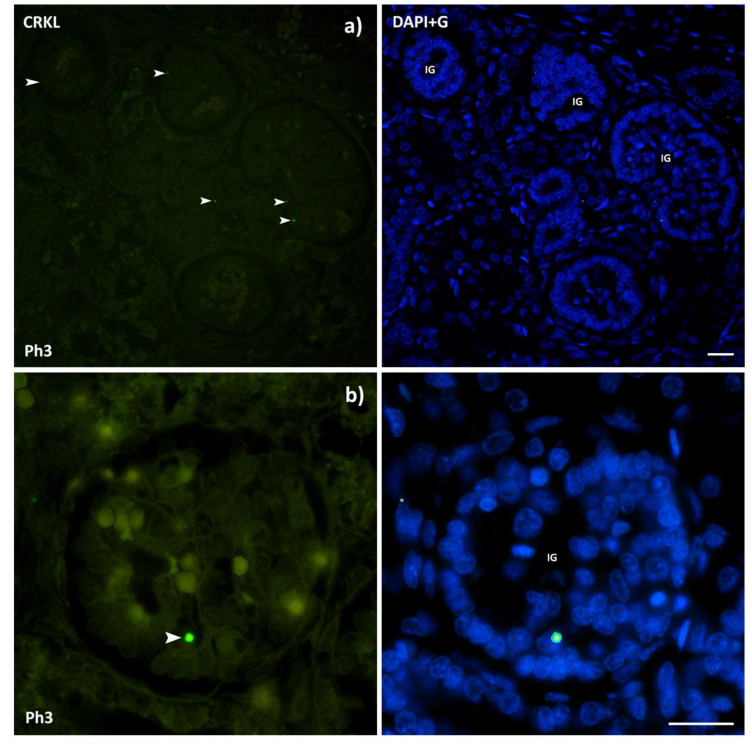
Immunofluorescence staining of human fetal kidneys with the CRKL (green) marker and the isolated signal with DAPI nuclear staining. Expression of CRKL (arrows), immature glomeruli (IG). The CRKL positive cells displayed strong punctate staining with mainly a single positive cell in a structure with the signal being localized within the nucleus. Image (**a**) was taken on magnification 40×, while (**b**) was taken on 100×. Scale bar is 20 μm.

The CRKL positive cells displayed strong punctate staining with mainly a single positive cell in a structure (Figure 10). Positive cells were observed in glomeruli, DCTs, PCTs, and all developmental structures with the signal being localized within the nucleus. There were not enough positive structures to implement cell counting with statistical significance, but the area percentage was calculated for all phases. The analysis showed statistically significant differences between Ph1 and Ph3 (*p* < 0.05), and Ph2 and Ph3 (*p* < 0.05) (Figure 3). The expression of CRKL proteins in tested human tissues was also ubiquitous, as demonstrated by qRT-PCR (Figure 4).

### 3.5. BCL2

The qRT-PCR analysis showed the expression of BCL2 mRNA in all tissues tested with no significant differences detected between the phases of development (Figure 4). A two-way ANOVA test with Tukey’s multiple comparisons test revealed no significant differences between AIFM3, AIF, and BCL2.

## 4. Discussion

This study aimed to investigate the spatio-temporal expression of three potential CAKUT candidate genes *CRKL*, *UBASH3A*, and *AIFM3* on rare samples of developing and early postnatal human kidneys, while *AIF* was included due to its protein’s homology with AIFM3, and *BCL2* was analyzed as a well-known anti-apoptotic factor.

Suboptimal conditions during organ development can lead to an increased risk of disease later in life. It is important to keep in mind that input signals rely on the attainability of extracellular stimuli and the condition of the cells, as well as on the status of proliferation and/or differentiation and other temporal and spatial aspects of the physiological and metabolic state of the cells. Thus, signals in the cellular environment and with them the expression of cell proteins is subject to a dynamic and flexible flow. Our study showed statistically significant differences in the temporal expression patterns of various markers, depending on the investigated developmental stage.

The *CRKL* gene has previously been recognized as an important factor during normal maturation of human kidneys, as well as in the appearance of DiGeorge syndrome due to the lack of differentiation and migration of kidney cells [16,24]. By now, the CRKL protein has been shown in different expression locations of the kidneys and other urinary tract structures of developing humans and in children [16]. Developing mouse kidneys showed expression of *Crkl* mostly in the ureteric bud related structures, while genetic inactivation of *Crkl* in the mouse model resulted in congenital kidney and urinary tract anomalies that resemble human CAKUT [16]. Our study showed limited, but strong expression of *CRKL* in developing kidneys, with increasing expression up to the third phase, the period where the majority (60%) of nephrons are formed. The fact that *CRKL* mRNA and proteins were detected in all examined phases of kidney development implies its role in maintenance of overall homeostasis and in maturation of kidney structures, continuing into the postnatal period.

CRKL is downstream of the scaffold protein DAB1 in the Reelin signaling pathway, where it has an essential function in neuronal positioning in the developing brain [43]. Our previous studies reported high *DAB1* expression in human fetal kidneys and, most recently, Dab1 −/− mice as potential novel CAKUT models as a result of renal hypoplasia in conjunction with foot process effacement of the podocytes [44,45]. Podocyte-specific *Crk1/2* and *Crkl* double-null mice displayed progressive albuminuria and abnormally long podocyte foot processes, but showed no tubular abnormalities, Crkl was located mostly in the podocyte cell bodies and primary processes and was suggested to be necessary for the proper formation and remodeling of mice podocyte foot processes [18]. Taking into account this information, the observed CAKUT phenotype in Dab1 −/− mice is presumably linked to inadequate CRKL activation. Observation of microphotographs depicted that the protein did not discriminate, location wise. However, the glomeruli seem to be the primary location of the detected signal. Further research is planned to narrow down the specific cells in the glomeruli, which is located using specific markers. In a study of changes in global gene expression patterns during development and maturation of the rat kidney, the authors state that genes that peaked in mid-embryogenesis, not unlike *CRKL* in our study, significantly covered those who control the spatiotemporal distribution [46]. This is in line with the known role of *CRKL* in the migration of kidney cells and the fact that nephrogenesis culminates in Ph3 where the maximum area percentage was described. Regardless of a detailed grasp on interactions that occur between CRKL and its substrates, there is still a shortage of information as to how they are integrated in real time and space.

The expression of *AIFM3* has been studied by Xie et al. on different human tissues, including kidneys. Their results, not unlike ours, demonstrated AIFM3 to be expressed mainly in the tubules, whereas weak expression in the glomeruli had been observed as well [19]. Our attained results demonstrated the appearance of modest glomerular staining in Ph4 with an inclination towards greater positive cell coverage over time. Although the kidneys of neonates have a full set of nephrons at birth, there are many developmental changes that occur during the first months and years of life [4,47]. The visible presence of AIFM3 in the glomeruli could be tied to its likely role in energy metabolism due to the four-fold increase of the GFR during this period as a result of the enlargement of the glomerular surface area. An increase in solute and water reabsorption in transport designated tubuli follows the GFR as a consequence of glomerulotubular balance [48]. The calculated raw scores imply that the DCTs convey the impression of stability during kidney development. On the other hand, the PCTs had a sudden increase of immunoreactivity in the cytoplasm upon entering Ph4. This can be attributed to an abundance of elongated mitochondria in the cytoplasm between basolateral infoldings and interdigitations, which are responsible for cellular respiration [49]. Multiple studies on rabbits demonstrated a developmental increase in transport and transporter abundance for most sodium-dependent transporters (particularly the sodium phosphate cotransporters) present on the brush border of the PCT, but also basolateral membrane transport around the time nephrogenesis ceases [50,51]. Schmidt and Horster measured the activity of Na-K ATPase in mature and immature PCTs and observed it to be doubled in mature tubules [52,53]. All of these results correspond to our examined Ph4. Therefore, our findings can imply great importance of *AIFM3* in the production of energy in the glomeruli and PCTs during nephrogenesis and tubular maturation on behalf of its known role in mitochondrial bioenergetics.

The most substantial and erratic expression was observed in *AIF*, which promotes the assembly of complex I of the electron transport chain in the mitochondria, a physiologically and pathologically relevant ROS-forming site [31]. Normal byproducts of mitochondrial metabolism essential for fetal development include ROS and their accumulation can reach potentially damaging levels, which must be normalized to prevent adverse offspring outcomes [54,55]. The importance of AIF as a redox-active enzyme was demonstrated using AIF-deficient mice which had an excessive production of ROS than can lead to oxidative stress, which plays a meaningful role in developmental origins of kidney disease [20]. The significantly stronger expression of AIF in the first three phases points to its decisive role in the fate of the cells, but only in mature convoluted tubules.

The significant homology and similar functions of AIFM3 and AIF imply that the housekeeping non-apoptogenic role of the maintenance of complex I under physiological conditions, as well as scavenging free radicals to promote cell survival may be shared by AIFM3 despite their modest co-localization. A study conducted on medaka nerve cells offered evidence of AIFM3 participating in proper development and maintenance of the nervous system by means of radical scavenging [21]. We believe the same could be applied to kidney development due to the considerable AIFM3 expression we observed in nephron developmental structures. The presence of AIFM3, including several scavenging molecules, detoxifies the excess ROS, avoiding the state of oxidative stress. A group of genes with the similar global gene expression pattern in the developing rat kidney as *AIFM3* in human samples, peaking in the neonatal period, included a significant number of genes involved in protection against oxidative stress and energy metabolism, which we believe to be the main roles of *AIFM3* [46].

The vast expression in the developing structures of the nephron and the fact that caspase inhibition was shown to halt development of the metanephros indicates the important involvement of AIFM3 in caspase-3 mediated apoptosis in early nephrogenesis [32,56]. On the other hand, the anti-apoptotic *BCL2* exhibited a steady expression through-out all the qRT-PCR examined phases, with comparable ΔCt results to the examined apoptosis inducing factors, even though they have contradicting roles in apoptosis which points to caspase-dependent apoptosis being a necessary, but secondary role of *AIFM3* in the developing kidney. This study leads us to conclude that *AIFM3* and *AIF* are important for promoting cell survival, but only *AIFM3* is considered a CAKUT candidate gene due to the lack of *AIF* expression in nephron developmental structures.

This is the first instance in which *UBASH3A* has been investigated in developing human kidneys from the 13th week to the 1st year and a half postnatal. The protein expression was thought to be more or less lymphoid specific, but our study confirmed its expression in the kidneys, as well. The analyzed structures of stained samples exhibited insignificant fluctuation of cell immunoreactivity between phases, while the analysis of area percentage found a markedly vaster expression of its protein in the prenatal Ph4 where maximum growth of the fetus is achieved. In vitro gene knockdown studies on a renal cell carcinoma cell line concluded that knockdown of *UBASH3A* inhibited cell migration and viability and therefore could induce metastasis in clear cell renal cell carcinoma [38]. These findings could perhaps help tie together the link between *UBASH3A* and CAKUT. Even though the regulatory effect of *UBASH3A* is not yet well understood, regulation of Syk, a protein tyrosine kinase, in cells where it plays a role in receptor signal transduction is the best characterized effect of TULA-family proteins on signaling [57]. Through its upregulation of Syk it may play a role in kidney development through the GDNF/Ret and PI3K pathways, which are important in the aspect of normal branching morphogenesis. Syk also ties it together with the Reelin signaling pathway which includes CRKL. What is certain about UBASH3A is that it is a Cbl-Interacting protein and was found to inhibit Cbl to suppress EGFR signaling [58]. Since it has been known for decades that the CRKL SH2 domain binds specifically to Cbl [59], it is also within the realm of possibility that CRKL and UBASH3A share a common pathway such as the EGFR signaling pathway, which would explain the resemblance of their expression patterns. Similar to *CRKL*, *UBASH3A* peaked in mid-embryogenesis which, according to the study of changes in global gene expression patterns during development and maturation of the rat kidney, would group it together with the genes that control spatiotemporal distribution [46].

UBASH3A exerts a pro-apoptotic effect which is mediated by AIF [19], but since AIF was absent from glomeruli as well as immature structures, this effect could take place only in the PCTs and DCTs. Possibly, the UBASH3A protein interacts with AIFM3 at least indirectly due to significant similarities with AIF. However, the modest colocalization of the apoptosis inducing factors could beg to differ. Further studies including double immunofluorescence with UBASH3A and AIFM3 are needed for further understanding of these signal cascades. Most importantly, the raw scores showed significantly greater immunoreactivity in the developing structures than mature ones, which would point to a meaningful role in nephrogenesis.

## 5. Conclusions

In conclusion, with advances in mouse research, multiple genes that critically regulate renal differentiation have been identified, but also some of these genes are not displayed in the mouse at all, or they have different paralogs. Therefore, even a limited number of human samples that were very difficult to acquire in our study, could still help elucidate the normal expression pattern of CAKUT candidate genes. In early kidney development, genes involved in protein translation and DNA replication are highly expressed. This would explain why, even though our suggested CAKUT candidate genes showed expression in the first phase, it was always significantly lesser than that of their main phase owing to their alternative roles.

Therefore, the abundant presence in different structures of the developing human kidney and dynamics of their expression found in this study indicate that *CRKL*, *UBASH3A*, and *AIFM3* might have important roles as renal development control genes. Further work will be needed to assess and reveal their function in regulating kidney differentiation. It is our hope that novel gene discovery will lead to better understanding of the genetic bases of kidney defects that will lead to improving of diagnostics and treatment within a precision medicine approach and even prevention of developmental origins of kidney disease.

## Figures and Tables

**Table 1:** Primers used in qRT-PCR.

Gene	Forward Primer	Reverse Primer
*CRKL*	5′-AGC AAT CCA GAA AAG AGT ACC C-3′	5′-TTC ACT TCG CCT TCC CAC-3′
*AIFM3*	5′-CAA CCG CAA AGT GAACAT TCC-3′	5′-TCC AGA GGT AGG GCA CAG-3′
*AIF*	5′-AAG CAG GCTCTA ACA TCT GG-3′	5′-TTC TCC AGC CAA TCT TCC AC-3′
*BCL2*	5′-GTG GAT GAC TGA GTA CCT GAA C-3′	5′-GCC AGG AGA AAT CAA ACA GAG G-3′
*UBASH3A*	5′-GAA TGG ACA AAA TGG GAA GCT G-3′	5′-TGT ACT CCT GGT AGC TCT CG-3′
*RPS9*	5′-GGA TTT CTT AGA GAG ACG CCT G-3′	5′-GGA CAA TGA AGG ACG GGA TG-3′

## Data Availability

Data is available per request.
